# The Anthelmintic Virtues of Kousso

**Published:** 1851-05

**Authors:** 


*The Anthelmintic virtues of Kousso.*—Numerous cases are reported in the late British journals, of the successful expulsion of tape worms by the use of Kousso, (Brazera anthelmintica.)

